# “Partner”, “Caregiver”, or “Co-Survivor”—Might the Label We Give the Partners of Cancer Patients Affect the Health Outcome of the Patients and Their Partners?

**DOI:** 10.3390/curroncol29010010

**Published:** 2021-12-27

**Authors:** Hannah M. K. McGillivray, Elisabetta E. L. Piccolo, Richard J. Wassersug

**Affiliations:** 1 Edinburgh Medical School, University of Edinburgh, Edinburgh EH16 4SB, UK; hannahmcgillivray99@gmail.com; 2 Independent Scholar, Surrey, BC V4A 9T5, Canada; e.piccolo01@gmail.com; 3 Department of Cellular and Physiological Sciences, Faculty of Medicine, The University of British Columbia, Vancouver, BC V6T 1Z3, Canada

**Keywords:** partner, caregiver, partnership, sexuality, quality of life, survival, counselling

## Abstract

Having a life partner significantly extends survival for most cancer patients. The label given to the partners of cancer patients may, however, influence the health of not just the patients but their partners. “Caregiver” is an increasingly common label for the partners of patients, but it carries an implicit burden. Referring to partners as “caregivers” may be detrimental to the partnerships, as it implies that the individuals are no longer able to be co-supportive. Recognizing this, there has been some effort to relabel cancer dyads as “co-survivors”. However, many cancer patients are not comfortable being called a “survivor”, and the same may apply to their partners. Cancer survivorship, we argue, could be enhanced by helping keep the bond between patients and their partners strong. This includes educating patients and partners about diverse coping strategies that individuals use when facing challenges to their health and wellbeing. We suggest that preemptive couples’ counselling in cancer centers may benefit both patients and their partners.

## 1. Introduction

One of the most established facts in cancer epidemiology—and, for that matter, most of medicine—is that having a long-term and stable spousal relationship means better outcomes for the patient. This was documented for 10 major cancers by Aizer et al. back in 2013, in his study of married patients [1]. Compared to patients without spouses, patients diagnosed with those cancers, who also had a partner, were more likely to be diagnosed earlier and to accept definitive treatment. They were also less likely to die as a result of their disease. For patients with many other cancers, too, the benefit of an intimate partner or spouse has been confirmed [2,3,4,5,6,7,8].

Here, we review labels that have been given to the partners of cancer patients and explore the idea that the labels themselves could impact the partners, the patients, and their partnership. We focus on what may be implicit but unstated messages in those labels. We suggest that healthcare providers be cautious about the labels they use in referring to the partners of patients and be attentive to the terms that are most helpful not just for the patient, but also for the dyad. We also offer strategies for helping couples maintain co-supportive partnerships in the face of cancer.

**Figure 1:**
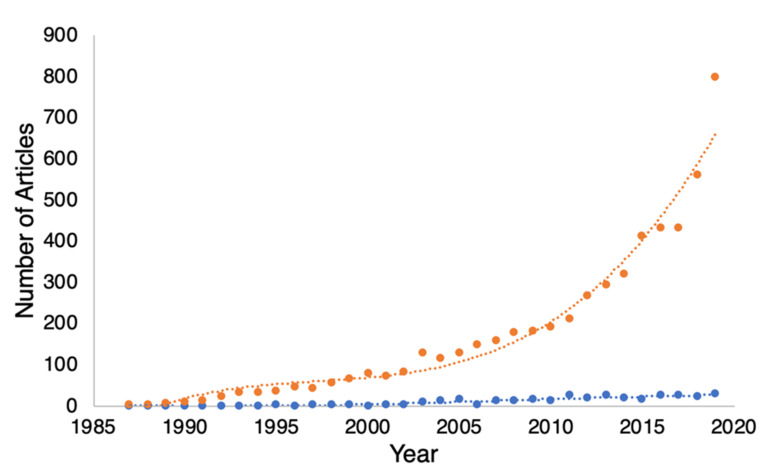
A graph of the number of papers listed in PubMed plotted against the year, which include the terms “cancer, patient, spouse, and caregiver” (orange dots) versus “cancer, patient, spouse and partner” (blue dots). The figure shows that over the last 30 years, reference to spousal “caregivers” has accelerated exponentially, while reference to “partners” has remained nearly flat. The curved lines fitted to the data are 3rd-order polynomials added simply as visual aids.

Clearly, many partners positively benefit from supporting their loved ones [9], however they are labelled. Currently, the most popular label for the partner of cancer patients within the psycho-oncology literature is “caregiver” (see Figure 1). In this essay, we proffer the idea that this label can influence the health and well-being of both individuals, as well as their dyadic relationship. We explore what costs may be associated with replacing the word “partner” with “caregiver”.

It should be noted that we are not referring to healthcare providers, nor to parents or guardians, who can all be caregivers under the broad umbrella of “support providers”. Rather, we are referring specifically to the long-term (i.e., spousal) partners of cancer patients.

The orange line in Figure 1 shows that “caregiver” is an increasingly popular term in the oncological setting. Part of the rise in this line reflects a genuine rise in the number of papers about cancer patients and their partners. We believe, however, that the rapid rise in papers that use the term “caregiver” rather than “partner” may reflect an uncritical acceptance of the term “caregiver” without considering the burden that that label might carry. Others before us have criticized the label “caregiver” in reference to the spousal partners of cancer patients. We discuss some of those concerns below. First, though, let us consider an alternative label that has been proposed for spousal dyads in the face of a serious illness. That term is “co-survivor”.

## 2. Is “Co-Survivor” a Better Term?

Recognizing the great emotional burden to spousal partners brought on by their partner’s cancer diagnosis, some authors have suggested replacing the couplets “patient and partner” and “patient and caregiver” with the term “co-survivors”. The term “co-survivor” used in this context is relatively new; it shows up in the oncological literature less than a half-dozen times before 2000.

The term, however, has not gained much popularity in this century. A Google Scholar search for “cancer” paired with the word “co-survivor(s)” (with or without the hyphen and with or without the “s”) yielded < 250 results as of October 2021. This pales in comparison to the 197,000 hits acquired with the search combination “cancer” and “caregiver(s)” (although that does not exclude professional healthcare providers).

Many cancer patients are themselves not comfortable with the label “survivor” [10,11,12]. In addition, patients and partners often differ in their concern about cancer reoccurrence [13] and, as such, would have different comfort levels with the term “survivor”. It is thus premature to assume the term “co-survivor” would have greater acceptance by patients and partners than the terms “patient” and “caregiver”. At the moment, we know of no data showing that either cancer patients or their partners have better health outcomes, dyadic adjustment, or individual quality of life when they are either referred to as “co-survivors” or label themselves as such.

Simply labelling a couple as “co-survivors” fails for three reasons. First, the term has never been in common use. Secondly, patients themselves may not be comfortable being labelled “survivors” and it is reasonable to suppose that that discomfort would carry over to their partners. Lastly, patients and partners may understand the word “survivor” to imply that a cancer that was life-threatening is being cured, and they may reject the term if they have different perspectives on those matters.

Labels that are used for any dyad can variously reflect similarities or differences between the individuals in the dyad. On one hand, the term “co-survivors” focuses on the equality and similarity of the individuals so labelled. On the other hand, “patient and caregiver” flags the inequality and dissimilarity between the individuals. “Patient and partner” is a more neutral term.

We favor the label “patient and partner” for its neutrality, but we recognize that serious illnesses and their treatments are stressful for both patients and their partners. As such, dealing with cancer can accentuate a couple’s shared concerns and bring partners closer together. Alternatively, though, major stressors can bring to the surface differences in how partners handle stress… and drive them apart.

## 3. Concordance and Contrast in Couples’ Coping Styles

How cancer and its treatments affect a couple’s dynamics often rests on how the individuals cope with stress. Recognizing and respecting differences in coping styles can be key to keeping a patient and partner co-supportive of each other and, thus, a strong dyad.

Several studies have documented that the stress of a cancer diagnosis can be greater on the partner than the patient, particularly for female partners [14]. Women report more distress than men regardless of whether the women are breast cancer patients or partners of prostate cancer patients [15].

Partners may have divergent and even conflicting adaptive strategies for dealing with illness, and this can challenge their partnership [16]. For example, a “monitor” might feel that it is a household priority to discuss and deliberate on the patient’s health, while a “blunter” would not want to over-analyze the situation [17]. (Male patients tend to be blunters [18].) If both partners appreciate and accept each other’s style, contrasting coping strategies can work out well [16], but when they argue over cancer-related concerns, their discord correlates with poorer patient health outcomes and poorer quality of life [17,19].

In the worst of situations, a partnership that was unstable before the cancer diagnosis can become unbearable for the patient, the partner, or both. This stress can lead to separation or divorce. That, in turn, has further negative consequences on the survival and quality of life for the patient. This has been documented in another Aizer-coauthored paper, which found that recently divorced cancer patients had lower cancer-specific survival than long-term married patients [20].

Aizer et al. (2013) pointed out that being married (i.e., having a partner) has more of a survival benefit than chemotherapy for five of the ten major cancers they examined [1]. Although not explicitly addressed by Aizer et al., a suggestion that follows from their data is that cancer centers might have the best outcomes if they had programs in place to help spousal couples stay as strong dyads when dealing with cancer. Couples’ counselling may sound tangential to cancer centers’ core mission, but we speculate that cancer centers could improve their survival statistics if they offered couples’ education or counselling as part of standards of care. The goal of such sessions would be to help partners recognize and respect each other’s coping style, to help them maintain a strong dyad when one of them is diagnosed with a cancer.

## 4. Sexual Intimacy between a Patient and a Caregiver

Sexuality is a stimulus for pair-bonding in our species and maintaining sexual intimacy gives couples cohesion. Although this has not been explored in any of the literature we have reviewed, we have some concern that labelling the partner of a cancer patient a “caregiver” could have negative implications to a couple’s sexual relationship. For “caregivers” who are not spouses but healthcare providers, it is inappropriate to have sexual relationships with patients because of the uneven power dynamic. Given that taboo, when a “patient and partner” are labelled “patient and caregiver”, we believe it suggests that they are either no longer sexual, or should not be sexual. Others have noted that there is a general social assumption that the chronically ill and disabled do not have sexual relationships, and the inequality implicit in the labels “patient” and “caregiver” bolsters that assumption [21,22]. Both the patient and the partner may pay a price for accepting the labels that carry this assumption. Indeed, loss of sexual intimacy has been documented as a major psychological stressor for spouses who were identified by researchers as the caregivers of cancer patients [16,17,18,20,23].

Over two-thirds of all patients with advanced cancer in a dyad identified by researchers as a “patient and spouse caregiver relationship” reported that “not feeling sexually attractive” was a moderate to severe problem for them [17,20]. Patients and partners may indeed have decreased sexual intimacy, but that would be a private matter. We thus believe the conjoint label “patient and caregiver” implies to others that there is little or no sexual intimacy in the relationship.

## 5. The Need to Keep Them Co-Supportive

With this in mind, we believe healthcare providers should recognize a couple as a partnership and treat them as a dyad if at all possible. That sounds simple and obvious, but it may be undermined by the language used to refer to couples in the cancer setting. Figure 1 shows how extensively the medical world has accepted the label “patient and caregiver” for what had previously been called “patient and partner”. The label “caregiver” may sound laudable, but it may, as suggested above, carry some subtle yet significant differences from the more neutral term “partner”.

We are not the first to recognize an implicit danger in labelling a patient’s partner as a “caregiver” (or more commonly in Europe, a “carer”). Henderson (2001) and others before us saw the label as detrimental to pre-existing relationships [24]. Molyneaux et al. [25] went further in criticizing the term “carer” because it “creates a division between people who might otherwise work together” with negative consequences to both health research and practice. Caregiving implies honorable self-sacrifice. However, for spouses, that label can be more of an albatross than an accolade [26,27]. Terms such as “spouse” and “partner” imply a dyad with some equal responsibilities between the spouses (i.e., the partners) to care for each other. When the dyad devolves into a “patient and caregiver” relationship, it is prima facie no longer an equal and co-supportive partnership; the caregiver carries the burden [28,29].

Based on the survivorship data reviewed above, cancer centers could be doing more to make sure that patients, who have a partner, have a good, strong and co-supportive relationship. Instead, the data in Figure 1 suggest that the oncology community has overwhelmingly accepted the label for the partners of patients as “caregivers” without critically assessing whether there might be costs to the patients and their partners in that label.

Prematurely labelling a patient’s partner as a “caregiver” may be prodromal to distress itself. A study of spousal caregivers of elderly cancer patients found that over 16% had clinical depression and 28% reported feeling distressed [30]. The researchers reported that caregiving negatively impacted spouses’ health. There is increasing awareness of the needs of patients’ partners who are truly performing in the role of caregivers [29]. Cancer centers should be praised for recognizing that the stress on those individuals can be even greater than the stress on the patients themselves [31,32]. In light of these facts, many cancer centers now offer psychosocial support for not just their patients, but also the spousal caregivers.

## 6. Strategies to Support Partnerships

Are there more specific ways for a “patient and partner” to keep their partnership strong, rather than presuming that it is, or should be, a “patient and caregiver” association in the face of illness? We believe that timely education can buffer couples from conflicts in coping when faced with cancer and other diseases. The best way for couples’ counsellors to protect couples from drifting apart is to have them informed about the diversity of coping strategies and willing to recognize that different strategies may serve the individual needs of the patient and the partner [16]. The value of couples’ education at the time of cancer diagnosis—by reducing distress for both patients and partners—has been demonstrated for educational programs offered to newly diagnosed prostate cancer patients and their partners [32,33,34,35,36,37]. Just as early education has been shown to help prostate cancer patients anticipate and manage the side effects of their drug treatments [38], early couples’ education may help cancer patients in general retain the benefits of a strong spousal dyad.

Educating both patients and partners about coping strategies for couples in the cancer setting can serve all couples facing a new cancer diagnosis. We invite cancer centers to offer a simple introductory seminar to new patients and their partners to educate them about the different ways that patients and partners often react to a cancer diagnosis. This would amount to preventative couples counselling. Such sessions do not need to be elaborate counselling but could include what we, as researchers and healthcare providers, know are the benefits of a strong partnership to long-term survival. Implementing such programs would fit well within the educational initiatives that are increasingly common in supportive care programs that are offered at the top-rated cancer centers in the industrial world.

It is important for both the cancer patient and the partner to be included in these programs so that they can both be informed about how the cancer diagnosis and treatments impact them individually. This would include educating them about diversity in coping strategies. Women, for example, are particularly good at noticing slight changes in their male partners’ health and demeanor. Men, in contrast, may strive to hide their distress or be simply unaware of how distressed or debilitated they actually appear. As documented by Kim et al. more than a decade ago, his poorer health or unrealistic response to his situation can raise her distress level [15]. An underappreciated and relevant finding of that study was that “women’s distress [from their awareness of their husband’s demeanor] predicted men’s physical health, over and above the men’s distress, …age, and cancer stage” [15]. Therefore, if we want to help the patients, we should be doing whatever we can to help their partners. These same concerns may relate to the same-sex partners of patients, but this has received relatively little research to date [39,40].

Such educational sessions could be introduced by letting attendees know that dealing with cancer is likely to be a challenge for both patients and partners, acknowledging their individual needs. The staff at the cancer center could then use that venue to find out how the couples view themselves. Inevitably, given individual differences in overall health status, some couples may very well fit the label “patient and caregiver” and be perfectly comfortable describing themselves as such. Others may not fit that dynamic nor like the label. However, either way, this frees the staff from having to guess or presume how the patient and the partner at that time view their dyad.

## 7. What More Can Be Done to Help Couples Stay as Couples in the Cancer Setting?

Increasingly, cancer centers are recognizing the benefit of exercise for both patients and partners. However, usually, these are promoted to meet the individual needs of the patient or caregiver. An alternative approach is to promote programs such as “Exercise Together” piloted at the Oregon Health and Science University [41]. This program trains patients and partners to be the fitness trainers for each other. It helps to affirm responsibility and support for patients and the partners concurrently, thus helping to strengthen the partnership and not just serving the patient or partner individually. Again, this program is likely to have the best buy-in when offered to the couple before the patient is challenged by treatment side effects. However, to the best of our knowledge, there are no published data on couples’ long-term survival and quality of life that consider the cost/benefit ratio for running such lifestyle programs within the cancer setting.

As cancers progress, partnerships will inevitably transition to a “patient and caregiver” relationship [42]. We accept that. However, we also contend that helping couples in the cancer setting before caregiving becomes medically, logistically, and emotionally unavoidable will benefit both the patient and the partner. In sum, we can do more to protect couples before cancer’s challenges become insurmountable and the couple’s status transitions to the patient/caregiver dynamic.

## 8. Conclusions

The terms “partner”, “caregiver”, or “co-survivor” may superficially seem similar, but they are not identical and may carry implicit costs to the couple. Research is warranted to find out more about who benefits or is burdened by being referred to as a “partner”, “caregiver”, or “co-survivor”.

We suggest that cancer centers may have better oncological outcomes overall if they address the needs of couples in a timely fashion. As noted by Molyneaux et al., medical care would improve if healthcare providers “acknowledge[d] pre-existing relationship[s] through the terminology they use” [25]. That starts with avoiding either antithetical label for the partners of cancer patients as “caregiver” or “co-survivor” unless they themselves so identify. From the perspective of institutional policy, staff can be encouraged not to address a person accompanying a patient as the patient’s “caregiver” unless they identify themselves that way. This is an easy policy for hospitals and clinics to put in place.

Although many spousal partnerships will end up as patient-and-caregiver partnerships, treating patients and the partners as co-supportive spouses is good cancer care because strong dyads mean better outcomes overall.
